# Poly[[tris­(μ-4,4′-bipyridine-κ^2^ *N*:*N*′)bis­(μ-l-lysinato-κ^3^ *N* ^1^,*O* ^1^:*O* ^1′^)dizinc(II)] tetra­nitrate 0.6-hydrate dimethyl­formamide disolvate]

**DOI:** 10.1107/S1600536812016121

**Published:** 2012-04-21

**Authors:** Shu-Qiang Li, Ning-Hai Hu

**Affiliations:** a Orthopaedic Department, First Hospital, Jilin University, Changchun 130021, People’s Republic of China; b Changchun Institute of Applied Chemistry, Chinese Academy of Sciences, Changchun 130022, People’s Republic of China

## Abstract

In the title compound, {[Zn_2_(C_6_H_14_N_2_O_2_)_2_(C_10_H_8_N_2_)_3_](NO_3_)_4_·0.6H_2_O·2C_3_H_7_NO}_*n*_, the Zn^II^ ion is six-coordinated with a distorted octa­hedral geometry by two carboxyl­ate O atoms and one amino N atom from two l-lysinate (l-lys) ligands, and three N atoms from three 4,4′-bipyridine (4,4′-bipy) ligands. The Zn^II^ ions are connected by the carboxyl­ate groups of the l-lys ligands in the *a*-axis direction and the bridging 4,4′-bipy ligands in the *b*- and *c*-axis directions, forming a three-dimensional cationic framework with channels along [100]. The nitrate anions and solvent water and dimethyl­formamide (DMF) mol­ecules are located in the channels and linked to the cationic framework by N—H⋯O and O—H⋯O hydrogen bonds. The occupancy of the water mol­ecule was fixed at 0.6. One of the DMF mol­ecules is disordered over two sets of sites, with an occupancy ratio of 0.632:0.368 (11).

## Related literature

For general background to the structures and properties of chiral coordination polymers, see: Dai *et al.* (2005 ▶); Kesanli & Lin (2003 ▶); Vaidhyanathan *et al.* (2006 ▶); Zaworotko (2001 ▶). For the structures of metal complexes with 4,4′-bipy and l-tyrosinate ligands, see: Li & Hu (2011 ▶); Zhang & Hu (2009 ▶). For the structures of Cu^II^ complexes with 4,4′-bipy and l-valinate ligands, see: Lou *et al.* (2007 ▶); Lou & Hong (2008 ▶). 
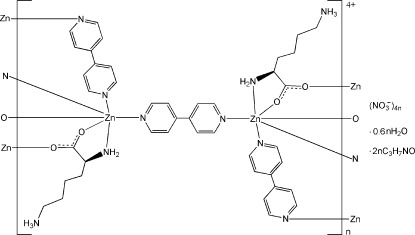


## Experimental

### 

#### Crystal data

[Zn_2_(C_6_H_14_N_2_O_2_)_2_(C_10_H_8_N_2_)_3_](NO_3_)_4_·0.6H_2_O·2C_3_H_7_NO
*M* *_r_* = 1296.76
Monoclinic, 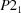

*a* = 10.3039 (4) Å
*b* = 24.9425 (10) Å
*c* = 11.5740 (4) Å
β = 93.197 (1)°
*V* = 2970.0 (2) Å^3^
*Z* = 2
Mo *K*α radiation
μ = 0.89 mm^−1^
*T* = 187 K
0.26 × 0.23 × 0.13 mm

#### Data collection

Bruker APEX CCD diffractometer
Absorption correction: multi-scan (*SADABS*; Sheldrick, 1996 ▶) *T*_min_ = 0.801, *T*_max_ = 0.893
16802 measured reflections
11087 independent reflections
10039 reflections with *I* > 2σ(*I*)
*R*_int_ = 0.019

#### Refinement

*R*[*F* ^2^ > 2σ(*F* ^2^)] = 0.041
*wR*(*F* ^2^) = 0.107
*S* = 1.00
11087 reflections
820 parameters
63 restraints
H-atom parameters constrained
Δρ_max_ = 0.81 e Å^−3^
Δρ_min_ = −0.33 e Å^−3^
Absolute structure: Flack (1983 ▶), 5102 Friedel pairs
Flack parameter: 0.003 (9)

### 

Data collection: *SMART* (Bruker, 2007 ▶); cell refinement: *SAINT* (Bruker, 2007 ▶); data reduction: *SAINT*; program(s) used to solve structure: *SHELXS97* (Sheldrick, 2008 ▶); program(s) used to refine structure: *SHELXL97* (Sheldrick, 2008 ▶); molecular graphics: *SHELXTL* (Sheldrick, 2008 ▶); software used to prepare material for publication: *SHELXTL*.

## Supplementary Material

Crystal structure: contains datablock(s) global, I. DOI: 10.1107/S1600536812016121/nk2149sup1.cif

Structure factors: contains datablock(s) I. DOI: 10.1107/S1600536812016121/nk2149Isup2.hkl

Additional supplementary materials:  crystallographic information; 3D view; checkCIF report

## Figures and Tables

**Table 1:** Hydrogen-bond geometry (Å, °)

*D*—H⋯*A*	*D*—H	H⋯*A*	*D*⋯*A*	*D*—H⋯*A*
O1*W*—H1*A*⋯O15	0.95	1.91	2.823 (11)	159
O1*W*—H1*B*⋯O10^i^	0.90	2.48	3.182 (8)	136
N7—H7*A*⋯O3^ii^	0.92	2.54	3.066 (4)	117
N7—H7*B*⋯O18	0.92	2.03	2.940 (9)	167
N7—H7*B*⋯O18′	0.92	2.32	3.215 (17)	163
N8—H8*A*⋯O17^iii^	0.91	1.86	2.755 (7)	166
N8—H8*B*⋯O8^iii^	0.91	2.04	2.842 (6)	147
N8—H8*B*⋯O9^iii^	0.91	2.39	3.057 (6)	130
N8—H8*C*⋯O5^iii^	0.91	2.00	2.907 (7)	172
N9—H9*A*⋯O1^iv^	0.92	2.44	2.999 (4)	119
N9—H9*B*⋯O10	0.92	2.16	3.075 (4)	174
N10—H10*A*⋯O14	0.91	2.00	2.869 (6)	160
N10—H10*A*⋯O16	0.91	2.44	3.183 (8)	139
N10—H10*B*⋯O11^v^	0.91	2.00	2.844 (5)	154
N10—H10*B*⋯O12^v^	0.91	2.48	3.066 (6)	123
N10—H10*C*⋯O7^i^	0.91	2.04	2.914 (7)	161

Symmetry codes: (i) 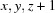; (ii) 
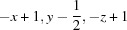
; (iii) 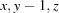; (iv) 
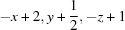
; (v) 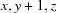.

**Fig. 1.**
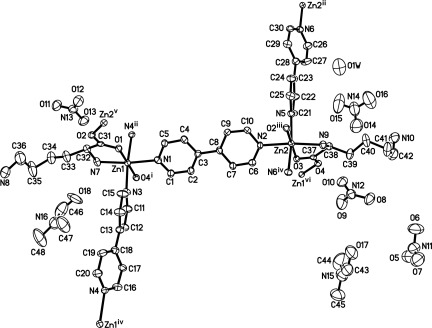
The asymmetric unit of (I). Displacement ellipsoids are drawn at the 50% probability level. H atoms and the minor component of the disordered DMF molecule have been omitted for clarity. [Symmetry codes: (i) 1 - x, -1/2 + y, 1 - z; (ii) x, y, 1 + z; (iii) 2 - x, 1/2 + y, 1 - z; (iv) x, y, -1 + z; (v) 2 - x, -1/2 + y, 1 - z; (vi) 1 - x, 1/2 + y, 1 - z.]

**Fig. 2.**
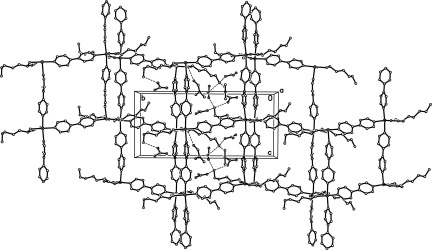
A view of the crystal packing of (I). H atoms have been omitted for clarity. Dashed lines denote hydrogen bonds.

## supplementary crystallographic information

### Comment

Chiral coordination polymers have attracted much interest because they exhibit potential applications in asymmetric catalysis and chiral separation (Kesanli & Lin, 2003). Mixed-ligand systems containing both chiral and achiral ligands have been developed as an effective approach to construct chiral complexes (Dai *et al.*, 2005; Vaidhyanathan *et al.*, 2006; Zaworotko, 2001). Amino acids are a kind of candidate for chiral building blocks, with their amino and carboxylate groups binding to metal ions (Lou *et al.*, 2007; Lou & Hong, 2008). We previously reported a chiral one-dimensional Zn(II) complex, [Zn(*L*-tyr)(4,4'-bipy)_2_(H_2_O)]NO_3_.2H_2_O, (II), (*L*-tyr = *L*-tyrosinate, 4,4'-bipy = 4,4'-bipyridine) (Li & Hu, 2011) and a chiral two-dimensional Cu(II) complex, [Cu_2_(*L*-tyr)_2_(4,4'-bipy)(NO_3_)_2_(H_2_O)_2_], (III), (Zhang & Hu, 2009). Herein, we present the title compound, (I), a three-dimensional Zn(II) complex with *L*-lysinate (*L*-lys) and 4,4'-bipy ligands.

In (I), the Zn(II) ion is six-coordinated by one N atom and two O atoms from two *L*-lys ligands, three N atoms from three 4,4'-bipy ligands in a distorted octahedral geometry (Fig. 1). The *L*-lys ligands bind to the Zn atoms in a µ-(κ^3^*N*,*O*:*O*') mode, the same as that observed in (II) and (III). The 4,4'-bipy ligands adopt a bridging mode, similar to that in (III) but different from the monodentate terminal mode in (II). The 4,4'-bipy ligands bridge the Zn atoms in the *b* and *c* directions, while the *L*-lys ligands bridge adjacent Zn atoms through the carboxylate groups in the *a* direction, forming a three-dimensional chiral cationic framework, which exhibits channels in the *a* direction (Fig. 2). The nitrate anions and the water and dimethylformamide (DMF) solvent molecules are located in the channels. The ammonium tails of the *L*-lys ligands extend into the channels and form N—H···O hydrogen bonds with the nitrate anions and DMF molecules (Table 1). Moreover, the water molecules and α-amino groups acting as donors form O—H···O and N—H···O hydrogen bonds with the nitrate anions, carboxylate groups and DMF molecules.

### Experimental

Zn(NO_3_)_2_.6H_2_O (0.119 g, 0.4 mmol), *L*-lysine (0.058 g, 0.4 mmol) and 4,4'-bipy (0.062 g, 0.4 mmol) were dissolved in water/DMF (20 ml, *v*/*v* 1:1) under stirring. The resulting solution was allowed to stand at room temperature and colorless crystals suitable for X-ray diffraction analysis were obtained after two weeks.

### Refinement

One of the DMF molecules is disordered over two sets of sites, with an occupancy ratio of 0.632 (11):0.368 (11). The water molecule is partly occupied. The occupancy factor was initially refined to 0.612 (11) and it was fixed at 0.60 in the final refinement. H atoms of the water molecule were located in a difference Fourier map and refined as riding atoms, with *U*_iso_(H) = 1.5*U*_eq_(O). Other H atoms were positioned geometrically and refined as riding atoms, with C—H = 0.95 (aromatic), 0.99 (CH_2_), 1.00 (CH) and 0.98 (CH_3_) Å and N—H = 0.92 (NH_2_) and 0.91 (NH_3_) Å and with *U*_iso_(H) = 1.2*U*_eq_(C, N).

### Crystal data

**Table:**

[Zn_2_(C_6_H_14_N_2_O_2_)_2_(C_10_H_8_N_2_)_3_](NO_3_)_4_·0.6H_2_O·2C_3_H_7_NO	*F*(000) = 1352
*M**_r_* = 1296.76	*D*_x_ = 1.450 Mg m^−^^3^
Monoclinic, *P*2_1_	Mo *K*α radiation, λ = 0.71073 Å
Hall symbol: P 2yb	Cell parameters from 7390 reflections
*a* = 10.3039 (4) Å	θ = 2.4–26.0°
*b* = 24.9425 (10) Å	µ = 0.89 mm^−^^1^
*c* = 11.5740 (4) Å	*T* = 187 K
β = 93.197 (1)°	Block, colorless
*V* = 2970.0 (2) Å^3^	0.26 × 0.23 × 0.13 mm
*Z* = 2	

### Data collection

**Table:**

Bruker APEX CCD diffractometer	11087 independent reflections
Radiation source: fine-focus sealed tube	10039 reflections with *I* > 2σ(*I*)
Graphite monochromator	*R*_int_ = 0.019
φ and ω scans	θ_max_ = 26.0°, θ_min_ = 1.6°
Absorption correction: multi-scan (*SADABS*; Sheldrick, 1996)	*h* = −11→12
*T*_min_ = 0.801, *T*_max_ = 0.893	*k* = −29→30
16802 measured reflections	*l* = −13→14

### Refinement

**Table:**

Refinement on *F*^2^	Secondary atom site location: difference Fourier map
Least-squares matrix: full	Hydrogen site location: inferred from neighbouring sites
*R*[*F*^2^ > 2σ(*F*^2^)] = 0.041	H-atom parameters constrained
*wR*(*F*^2^) = 0.107	*w* = 1/[σ^2^(*F*_o_^2^) + (0.0664*P*)^2^] where *P* = (*F*_o_^2^ + 2*F*_c_^2^)/3
*S* = 1.00	(Δ/σ)_max_ = 0.013
11087 reflections	Δρ_max_ = 0.81 e Å^−^^3^
820 parameters	Δρ_min_ = −0.33 e Å^−^^3^
63 restraints	Absolute structure: Flack (1983), 5102 Friedel pairs
Primary atom site location: structure-invariant direct methods	Flack parameter: 0.003 (9)

### Fractional atomic coordinates and isotropic or equivalent isotropic displacement parameters (Å^2^)

**Table:**

	*x*	*y*	*z*	*U*_iso_*/*U*_eq_	Occ. (<1)
Zn1	0.56823 (4)	0.220094 (14)	0.41441 (3)	0.01985 (10)	
Zn2	0.93007 (3)	0.646779 (14)	0.56498 (3)	0.01945 (10)	
O1	0.7620 (2)	0.19164 (10)	0.4199 (2)	0.0224 (5)	
O2	0.8839 (2)	0.11711 (10)	0.4272 (2)	0.0223 (5)	
O3	0.7372 (2)	0.67874 (10)	0.5549 (2)	0.0214 (5)	
O4	0.6204 (2)	0.75003 (10)	0.5995 (2)	0.0226 (6)	
N1	0.6347 (3)	0.30147 (14)	0.4389 (3)	0.0247 (7)	
N2	0.8485 (3)	0.56755 (12)	0.5548 (2)	0.0213 (6)	
N3	0.5749 (3)	0.23036 (15)	0.2219 (3)	0.0285 (8)	
N4	0.5693 (3)	0.21814 (15)	−0.3919 (2)	0.0232 (6)	
N5	0.9354 (3)	0.63940 (14)	0.7606 (2)	0.0241 (7)	
N6	0.9337 (3)	0.64466 (16)	1.3743 (2)	0.0234 (6)	
N7	0.5354 (3)	0.13638 (14)	0.4023 (3)	0.0301 (8)	
H7A	0.4746	0.1266	0.4537	0.036*	
H7B	0.5024	0.1281	0.3290	0.036*	
N8	0.7276 (5)	−0.09001 (18)	0.1681 (4)	0.0660 (13)	
H8A	0.6545	−0.1075	0.1411	0.079*	
H8B	0.7932	−0.1141	0.1809	0.079*	
H8C	0.7509	−0.0655	0.1148	0.079*	
N9	0.9625 (3)	0.73065 (13)	0.5758 (3)	0.0254 (7)	
H9A	1.0364	0.7373	0.6219	0.030*	
H9B	0.9750	0.7441	0.5032	0.030*	
N10	0.8106 (4)	0.94545 (17)	0.7743 (4)	0.0570 (11)	
H10A	0.8015	0.9123	0.8058	0.068*	
H10B	0.7364	0.9646	0.7819	0.068*	
H10C	0.8786	0.9627	0.8116	0.068*	
C1	0.5721 (4)	0.34315 (16)	0.3901 (3)	0.0316 (9)	
H1	0.5006	0.3366	0.3365	0.038*	
C2	0.6079 (4)	0.39617 (16)	0.4148 (3)	0.0312 (9)	
H2	0.5605	0.4249	0.3790	0.037*	
C3	0.7122 (4)	0.40660 (15)	0.4913 (3)	0.0232 (8)	
C4	0.7785 (4)	0.36297 (16)	0.5417 (3)	0.0286 (9)	
H4	0.8515	0.3684	0.5942	0.034*	
C5	0.7360 (3)	0.31163 (15)	0.5139 (3)	0.0255 (8)	
H5	0.7807	0.2821	0.5496	0.031*	
C6	0.7558 (4)	0.55603 (16)	0.4730 (3)	0.0274 (8)	
H6	0.7195	0.5847	0.4280	0.033*	
C7	0.7106 (4)	0.50566 (15)	0.4503 (3)	0.0274 (8)	
H7	0.6470	0.4998	0.3890	0.033*	
C8	0.7575 (3)	0.46277 (15)	0.5168 (3)	0.0237 (8)	
C9	0.8495 (4)	0.47439 (16)	0.6051 (3)	0.0319 (9)	
H9	0.8823	0.4467	0.6549	0.038*	
C10	0.8935 (4)	0.52661 (17)	0.6205 (3)	0.0299 (9)	
H10	0.9583	0.5337	0.6801	0.036*	
C11	0.4679 (4)	0.24046 (18)	0.1545 (3)	0.0337 (10)	
H11	0.3899	0.2483	0.1912	0.040*	
C12	0.4639 (4)	0.24026 (17)	0.0350 (3)	0.0319 (9)	
H12	0.3845	0.2469	−0.0084	0.038*	
C13	0.5770 (4)	0.23018 (17)	−0.0211 (3)	0.0290 (10)	
C14	0.6897 (4)	0.2229 (2)	0.0483 (3)	0.0358 (9)	
H14	0.7709	0.2186	0.0143	0.043*	
C15	0.6835 (4)	0.2221 (2)	0.1664 (3)	0.0365 (9)	
H15	0.7615	0.2152	0.2117	0.044*	
C16	0.4819 (4)	0.24829 (18)	−0.3386 (3)	0.0332 (10)	
H16	0.4180	0.2670	−0.3851	0.040*	
C17	0.4805 (4)	0.25351 (17)	−0.2188 (3)	0.0296 (9)	
H17	0.4164	0.2748	−0.1848	0.035*	
C18	0.5756 (4)	0.22671 (19)	−0.1495 (3)	0.0267 (9)	
C19	0.6641 (4)	0.19624 (18)	−0.2039 (3)	0.0358 (10)	
H19	0.7296	0.1772	−0.1599	0.043*	
C20	0.6575 (4)	0.19326 (18)	−0.3237 (3)	0.0349 (10)	
H20	0.7205	0.1720	−0.3592	0.042*	
C21	0.8295 (4)	0.6508 (2)	0.8174 (3)	0.0362 (9)	
H21	0.7514	0.6586	0.7731	0.043*	
C22	0.8265 (4)	0.6521 (2)	0.9363 (3)	0.0351 (9)	
H22	0.7488	0.6619	0.9714	0.042*	
C23	0.9371 (4)	0.63896 (17)	1.0046 (3)	0.0270 (9)	
C24	1.0445 (4)	0.62388 (17)	0.9460 (3)	0.0314 (9)	
H24	1.1211	0.6121	0.9880	0.038*	
C25	1.0411 (4)	0.62576 (17)	0.8277 (3)	0.0302 (9)	
H25	1.1181	0.6169	0.7906	0.036*	
C26	0.8586 (4)	0.67658 (18)	1.3066 (3)	0.0361 (10)	
H26	0.8032	0.7011	1.3428	0.043*	
C27	0.8563 (4)	0.67624 (19)	1.1882 (3)	0.0408 (11)	
H27	0.8010	0.7001	1.1445	0.049*	
C28	0.9368 (4)	0.64021 (18)	1.1321 (3)	0.0269 (8)	
C29	1.0147 (4)	0.60713 (18)	1.2027 (3)	0.0296 (9)	
H29	1.0700	0.5817	1.1694	0.035*	
C30	1.0119 (4)	0.61110 (17)	1.3200 (3)	0.0275 (8)	
H30	1.0687	0.5888	1.3661	0.033*	
C31	0.7762 (3)	0.14177 (17)	0.4243 (3)	0.0212 (7)	
C32	0.6570 (3)	0.10546 (16)	0.4278 (3)	0.0268 (8)	
H32	0.6540	0.0922	0.5091	0.032*	
C33	0.6704 (4)	0.05621 (17)	0.3512 (4)	0.0421 (11)	
H33A	0.6645	0.0680	0.2694	0.051*	
H33B	0.7583	0.0409	0.3670	0.051*	
C34	0.5725 (5)	0.0125 (2)	0.3652 (5)	0.0540 (13)	
H34A	0.4840	0.0281	0.3616	0.065*	
H34B	0.5881	−0.0046	0.4419	0.065*	
C35	0.5818 (6)	−0.0303 (2)	0.2692 (6)	0.0812 (19)	
H35A	0.5065	−0.0548	0.2719	0.097*	
H35B	0.5765	−0.0122	0.1929	0.097*	
C36	0.7015 (6)	−0.0617 (3)	0.2801 (6)	0.0804 (18)	
H36A	0.6945	−0.0886	0.3423	0.096*	
H36B	0.7754	−0.0378	0.3021	0.096*	
C37	0.7262 (3)	0.72627 (15)	0.5884 (2)	0.0175 (7)	
C38	0.8500 (3)	0.75806 (14)	0.6249 (3)	0.0239 (8)	
H38	0.8622	0.7542	0.7108	0.029*	
C39	0.8394 (4)	0.81792 (16)	0.6008 (4)	0.0371 (10)	
H39A	0.8387	0.8239	0.5162	0.045*	
H39B	0.7559	0.8312	0.6279	0.045*	
C40	0.9493 (5)	0.84975 (19)	0.6590 (5)	0.0478 (12)	
H40A	1.0329	0.8332	0.6401	0.057*	
H40B	0.9428	0.8475	0.7439	0.057*	
C41	0.9503 (5)	0.90914 (19)	0.6232 (5)	0.0618 (15)	
H41A	1.0274	0.9265	0.6620	0.074*	
H41B	0.9606	0.9110	0.5387	0.074*	
C42	0.8355 (6)	0.9400 (2)	0.6496 (5)	0.0609 (14)	
H42A	0.7586	0.9230	0.6098	0.073*	
H42B	0.8439	0.9764	0.6166	0.073*	
O1W	1.0534 (7)	0.7542 (3)	1.0804 (7)	0.094 (2)	0.60
H1A	0.9869	0.7577	1.0201	0.141*	0.60
H1B	1.0386	0.7803	1.1317	0.141*	0.60
N11	0.9419 (6)	0.9875 (2)	0.0281 (5)	0.0771 (15)	
O5	0.8211 (5)	0.9923 (2)	0.0153 (4)	0.0944 (15)	
O6	0.9935 (6)	0.9850 (2)	0.1267 (4)	0.121 (2)	
O7	1.0058 (5)	0.9887 (2)	−0.0591 (5)	0.0891 (14)	
N12	0.9215 (4)	0.81777 (17)	0.3054 (3)	0.0471 (10)	
O8	0.9535 (4)	0.86466 (16)	0.2816 (4)	0.0716 (12)	
O9	0.8025 (4)	0.80580 (18)	0.2939 (4)	0.0813 (14)	
O10	1.0025 (3)	0.78428 (14)	0.3412 (3)	0.0488 (8)	
N13	0.5965 (4)	0.04442 (16)	0.7044 (3)	0.0408 (9)	
O11	0.5668 (3)	−0.00369 (14)	0.7245 (3)	0.0572 (9)	
O12	0.7113 (3)	0.05824 (15)	0.7120 (3)	0.0551 (10)	
O13	0.5102 (3)	0.07704 (15)	0.6750 (3)	0.0550 (9)	
N14	0.8082 (6)	0.8281 (2)	0.9323 (6)	0.0852 (16)	
O14	0.7168 (5)	0.8458 (2)	0.8613 (5)	0.1016 (16)	
O15	0.8287 (5)	0.7804 (2)	0.9411 (7)	0.136 (2)	
O16	0.8814 (6)	0.8622 (2)	0.9766 (6)	0.136 (2)	
O17	0.4854 (5)	0.8717 (2)	0.0970 (5)	0.115 (2)	
N15	0.2865 (5)	0.8676 (2)	0.0072 (4)	0.0790 (15)	
C43	0.4144 (5)	0.8691 (3)	0.0078 (6)	0.0826 (19)	
H43	0.4541	0.8680	−0.0644	0.099*	
C44	0.2217 (8)	0.8683 (4)	0.1147 (6)	0.136 (4)	
H44A	0.2559	0.8392	0.1647	0.203*	
H44B	0.1281	0.8632	0.0988	0.203*	
H44C	0.2373	0.9027	0.1536	0.203*	
C45	0.2102 (7)	0.8662 (3)	−0.1010 (5)	0.106 (3)	
H45A	0.2651	0.8547	−0.1628	0.158*	
H45B	0.1756	0.9020	−0.1187	0.158*	
H45C	0.1381	0.8409	−0.0950	0.158*	
O18	0.4301 (14)	0.0931 (6)	0.1802 (10)	0.132 (6)	0.632 (11)
N16	0.4160 (12)	0.0515 (5)	0.0087 (9)	0.073 (4)	0.632 (11)
C46	0.3757 (11)	0.0613 (4)	0.1123 (9)	0.097 (5)	0.632 (11)
H46	0.3009	0.0430	0.1359	0.117*	0.632 (11)
C47	0.5301 (11)	0.0789 (5)	−0.0281 (10)	0.099 (5)	0.632 (11)
H47A	0.5649	0.1022	0.0344	0.148*	0.632 (11)
H47B	0.5067	0.1007	−0.0967	0.148*	0.632 (11)
H47C	0.5960	0.0525	−0.0469	0.148*	0.632 (11)
C48	0.340 (2)	0.0177 (10)	−0.0733 (13)	0.120 (9)	0.632 (11)
H48A	0.2504	0.0154	−0.0499	0.180*	0.632 (11)
H48B	0.3784	−0.0183	−0.0739	0.180*	0.632 (11)
H48C	0.3411	0.0333	−0.1509	0.180*	0.632 (11)
O18'	0.484 (2)	0.1047 (8)	0.1345 (15)	0.095 (8)	0.368 (11)
N16'	0.367 (2)	0.0472 (9)	0.0221 (11)	0.054 (5)	0.368 (11)
C46'	0.4563 (13)	0.0842 (6)	0.0395 (14)	0.063 (5)	0.368 (11)
H46'	0.5023	0.0960	−0.0247	0.076*	0.368 (11)
C47'	0.3019 (16)	0.0244 (8)	0.1188 (13)	0.097 (9)	0.368 (11)
H47D	0.3278	0.0440	0.1897	0.146*	0.368 (11)
H47E	0.3262	−0.0134	0.1280	0.146*	0.368 (11)
H47F	0.2075	0.0272	0.1038	0.146*	0.368 (11)
C48'	0.331 (3)	0.0274 (14)	−0.0941 (14)	0.110 (15)	0.368 (11)
H48D	0.3640	0.0521	−0.1516	0.166*	0.368 (11)
H48E	0.2365	0.0249	−0.1045	0.166*	0.368 (11)
H48F	0.3696	−0.0082	−0.1042	0.166*	0.368 (11)

### Atomic displacement parameters (Å^2^)

**Table:**

	*U*^11^	*U*^22^	*U*^33^	*U*^12^	*U*^13^	*U*^23^
Zn1	0.0190 (2)	0.0191 (2)	0.02137 (18)	0.00000 (16)	0.00039 (14)	−0.00195 (16)
Zn2	0.0203 (2)	0.0185 (2)	0.01949 (18)	−0.00060 (16)	0.00063 (14)	−0.00140 (16)
O1	0.0200 (12)	0.0209 (15)	0.0260 (13)	0.0019 (11)	−0.0007 (10)	−0.0023 (10)
O2	0.0192 (12)	0.0240 (15)	0.0236 (13)	0.0035 (10)	−0.0004 (10)	0.0007 (10)
O3	0.0201 (13)	0.0190 (14)	0.0249 (12)	−0.0006 (10)	−0.0017 (10)	−0.0005 (10)
O4	0.0190 (13)	0.0241 (15)	0.0246 (13)	0.0031 (10)	−0.0006 (10)	−0.0003 (10)
N1	0.0204 (16)	0.0278 (19)	0.0260 (16)	−0.0033 (13)	0.0010 (12)	−0.0022 (13)
N2	0.0237 (16)	0.0138 (16)	0.0261 (15)	0.0000 (13)	0.0000 (12)	−0.0007 (12)
N3	0.0278 (17)	0.036 (2)	0.0211 (15)	−0.0023 (15)	−0.0012 (12)	−0.0013 (14)
N4	0.0307 (16)	0.0216 (16)	0.0173 (13)	0.0035 (15)	0.0003 (11)	0.0008 (14)
N5	0.0266 (15)	0.0259 (19)	0.0199 (14)	−0.0017 (14)	0.0021 (11)	−0.0010 (13)
N6	0.0241 (15)	0.0269 (17)	0.0192 (14)	0.0011 (15)	0.0021 (11)	−0.0018 (15)
N7	0.0282 (16)	0.026 (2)	0.0362 (17)	−0.0057 (15)	0.0034 (13)	−0.0080 (13)
N8	0.077 (3)	0.047 (3)	0.072 (3)	0.024 (2)	−0.012 (2)	−0.008 (2)
N9	0.0231 (15)	0.023 (2)	0.0301 (16)	0.0003 (14)	0.0015 (12)	−0.0009 (12)
N10	0.070 (3)	0.042 (3)	0.058 (3)	0.019 (2)	0.005 (2)	−0.001 (2)
C1	0.033 (2)	0.022 (2)	0.039 (2)	−0.0016 (16)	−0.0107 (16)	0.0016 (16)
C2	0.029 (2)	0.021 (2)	0.042 (2)	−0.0020 (16)	−0.0104 (16)	0.0003 (16)
C3	0.0217 (18)	0.0210 (19)	0.0267 (18)	0.0017 (14)	0.0010 (14)	0.0004 (14)
C4	0.026 (2)	0.026 (2)	0.033 (2)	−0.0053 (16)	−0.0038 (15)	−0.0021 (16)
C5	0.024 (2)	0.019 (2)	0.033 (2)	0.0008 (15)	−0.0032 (15)	−0.0016 (15)
C6	0.026 (2)	0.025 (2)	0.030 (2)	0.0019 (16)	−0.0066 (15)	0.0000 (15)
C7	0.027 (2)	0.020 (2)	0.034 (2)	0.0017 (16)	−0.0065 (15)	−0.0033 (15)
C8	0.0217 (18)	0.0211 (19)	0.0280 (19)	−0.0027 (14)	−0.0017 (14)	−0.0037 (14)
C9	0.038 (2)	0.021 (2)	0.035 (2)	−0.0032 (16)	−0.0142 (17)	0.0056 (16)
C10	0.033 (2)	0.025 (2)	0.031 (2)	−0.0040 (16)	−0.0110 (16)	−0.0011 (16)
C11	0.037 (2)	0.045 (3)	0.0198 (19)	0.0031 (19)	0.0057 (16)	−0.0015 (17)
C12	0.027 (2)	0.042 (3)	0.027 (2)	0.0018 (17)	−0.0035 (16)	−0.0009 (17)
C13	0.037 (2)	0.031 (3)	0.0192 (18)	0.0016 (18)	0.0038 (15)	0.0005 (16)
C14	0.0264 (19)	0.057 (3)	0.0240 (18)	0.006 (2)	0.0041 (14)	0.001 (2)
C15	0.0234 (19)	0.060 (3)	0.0255 (18)	0.003 (2)	−0.0005 (14)	0.001 (2)
C16	0.035 (2)	0.043 (3)	0.0220 (19)	−0.0008 (19)	−0.0001 (16)	0.0011 (17)
C17	0.035 (2)	0.033 (2)	0.0207 (19)	0.0098 (18)	−0.0020 (15)	−0.0057 (16)
C18	0.0277 (19)	0.030 (2)	0.0223 (18)	−0.0017 (18)	0.0018 (14)	0.0004 (17)
C19	0.043 (2)	0.042 (3)	0.0222 (19)	0.0166 (19)	0.0005 (17)	0.0058 (17)
C20	0.040 (2)	0.040 (3)	0.025 (2)	0.0165 (19)	0.0025 (17)	−0.0013 (17)
C21	0.036 (2)	0.051 (3)	0.0211 (17)	0.008 (2)	−0.0032 (15)	0.002 (2)
C22	0.035 (2)	0.050 (3)	0.0201 (17)	0.009 (2)	0.0038 (14)	0.0045 (19)
C23	0.033 (2)	0.026 (2)	0.0219 (18)	0.0010 (17)	−0.0020 (14)	0.0005 (16)
C24	0.032 (2)	0.039 (3)	0.0224 (19)	0.0048 (17)	0.0001 (16)	−0.0022 (16)
C25	0.025 (2)	0.038 (3)	0.027 (2)	0.0022 (16)	0.0021 (15)	−0.0036 (16)
C26	0.050 (3)	0.036 (3)	0.0226 (19)	0.015 (2)	0.0023 (18)	−0.0023 (17)
C27	0.050 (3)	0.048 (3)	0.024 (2)	0.024 (2)	−0.0003 (18)	0.0052 (18)
C28	0.032 (2)	0.028 (2)	0.0213 (18)	0.0037 (18)	0.0032 (14)	−0.0024 (17)
C29	0.025 (2)	0.038 (2)	0.026 (2)	0.0027 (17)	0.0045 (15)	−0.0027 (17)
C30	0.027 (2)	0.029 (2)	0.0256 (19)	0.0068 (16)	−0.0046 (15)	−0.0010 (16)
C31	0.0264 (18)	0.026 (2)	0.0113 (14)	0.0009 (16)	−0.0003 (12)	0.0009 (14)
C32	0.0261 (19)	0.027 (2)	0.0272 (19)	−0.0018 (15)	0.0038 (15)	−0.0013 (15)
C33	0.034 (2)	0.033 (3)	0.059 (3)	−0.0053 (18)	0.005 (2)	−0.016 (2)
C34	0.056 (3)	0.036 (3)	0.070 (3)	−0.005 (2)	0.007 (3)	−0.009 (2)
C35	0.074 (4)	0.048 (3)	0.121 (6)	−0.010 (3)	0.001 (4)	−0.038 (3)
C36	0.078 (4)	0.060 (4)	0.100 (5)	0.006 (3)	−0.016 (4)	−0.021 (3)
C37	0.0177 (16)	0.023 (2)	0.0120 (14)	−0.0003 (15)	0.0011 (11)	0.0007 (13)
C38	0.0243 (18)	0.0209 (18)	0.0264 (19)	0.0007 (14)	0.0028 (15)	−0.0009 (14)
C39	0.034 (2)	0.025 (2)	0.054 (3)	0.0025 (17)	0.012 (2)	0.0017 (19)
C40	0.040 (3)	0.031 (3)	0.074 (3)	−0.009 (2)	0.017 (2)	−0.018 (2)
C41	0.084 (4)	0.033 (3)	0.072 (4)	−0.016 (2)	0.032 (3)	−0.005 (2)
C42	0.078 (4)	0.045 (3)	0.060 (3)	0.000 (3)	0.011 (3)	0.007 (2)
O1W	0.099 (5)	0.091 (6)	0.097 (6)	−0.014 (4)	0.046 (5)	−0.018 (4)
N11	0.110 (5)	0.055 (3)	0.064 (3)	0.008 (3)	−0.026 (3)	0.005 (3)
O5	0.120 (4)	0.089 (4)	0.072 (3)	0.025 (3)	−0.010 (3)	−0.005 (2)
O6	0.184 (6)	0.091 (4)	0.079 (3)	0.021 (4)	−0.066 (4)	−0.001 (3)
O7	0.082 (3)	0.088 (4)	0.096 (4)	−0.007 (3)	−0.009 (3)	0.023 (3)
N12	0.042 (2)	0.050 (3)	0.049 (2)	−0.016 (2)	−0.0059 (18)	0.0044 (19)
O8	0.055 (2)	0.056 (3)	0.100 (3)	−0.0173 (19)	−0.027 (2)	0.031 (2)
O9	0.038 (2)	0.063 (3)	0.142 (4)	−0.013 (2)	−0.014 (2)	0.013 (3)
O10	0.0351 (17)	0.052 (2)	0.058 (2)	−0.0029 (16)	−0.0070 (15)	0.0159 (17)
N13	0.035 (2)	0.043 (2)	0.043 (2)	−0.0078 (18)	−0.0074 (16)	0.0057 (17)
O11	0.049 (2)	0.041 (2)	0.080 (2)	−0.0115 (16)	−0.0111 (18)	0.0115 (17)
O12	0.0346 (19)	0.048 (2)	0.081 (3)	−0.0100 (16)	−0.0079 (17)	0.0069 (19)
O13	0.042 (2)	0.052 (2)	0.070 (2)	0.0007 (17)	−0.0129 (16)	0.0124 (18)
N14	0.083 (4)	0.059 (4)	0.113 (5)	−0.013 (3)	−0.002 (4)	−0.010 (3)
O14	0.096 (4)	0.097 (4)	0.111 (4)	−0.025 (3)	0.003 (3)	0.022 (3)
O15	0.104 (4)	0.062 (4)	0.240 (8)	−0.005 (3)	0.009 (4)	0.019 (4)
O16	0.147 (5)	0.115 (5)	0.147 (5)	−0.045 (4)	0.002 (4)	−0.033 (4)
O17	0.124 (4)	0.083 (4)	0.131 (5)	−0.014 (3)	−0.059 (4)	−0.007 (3)
N15	0.082 (4)	0.062 (3)	0.090 (4)	0.016 (3)	−0.017 (3)	−0.016 (3)
C43	0.081 (5)	0.062 (4)	0.102 (5)	0.014 (3)	−0.021 (4)	−0.004 (4)
C44	0.183 (9)	0.110 (7)	0.120 (7)	0.011 (6)	0.068 (7)	0.010 (6)
C45	0.107 (6)	0.107 (7)	0.098 (5)	0.016 (5)	−0.045 (5)	−0.007 (5)
O18	0.159 (13)	0.141 (13)	0.085 (8)	0.078 (9)	−0.080 (8)	−0.072 (9)
N16	0.108 (12)	0.055 (6)	0.057 (6)	0.000 (7)	0.015 (6)	0.001 (5)
C46	0.098 (10)	0.074 (9)	0.124 (13)	0.030 (7)	0.036 (9)	0.023 (9)
C47	0.112 (10)	0.094 (9)	0.091 (9)	−0.051 (8)	0.027 (8)	−0.025 (7)
C48	0.16 (2)	0.099 (12)	0.107 (13)	−0.079 (13)	0.045 (13)	−0.044 (11)
O18'	0.082 (12)	0.058 (9)	0.137 (19)	−0.033 (9)	−0.067 (11)	0.020 (11)
N16'	0.061 (11)	0.082 (11)	0.019 (7)	−0.010 (8)	0.008 (6)	−0.019 (7)
C46'	0.053 (10)	0.049 (10)	0.090 (14)	−0.001 (8)	0.016 (10)	−0.004 (9)
C47'	0.106 (17)	0.12 (2)	0.065 (11)	0.070 (15)	0.019 (11)	0.034 (12)
C48'	0.068 (17)	0.17 (4)	0.094 (19)	0.007 (18)	0.011 (14)	−0.09 (2)

### Geometric parameters (Å, º)

**Table:**

Zn1—O4^i^	2.080 (2)	C24—C25	1.368 (5)
Zn1—N7	2.119 (3)	C24—H24	0.9500
Zn1—O1	2.117 (2)	C25—H25	0.9500
Zn1—N1	2.156 (3)	C26—C27	1.369 (5)
Zn1—N4^ii^	2.242 (3)	C26—H26	0.9500
Zn1—N3	2.248 (3)	C27—C28	1.406 (6)
Zn2—O2^iii^	2.052 (2)	C27—H27	0.9500
Zn2—N9	2.121 (3)	C28—C29	1.385 (6)
Zn2—O3	2.139 (2)	C29—C30	1.363 (5)
Zn2—N2	2.148 (3)	C29—H29	0.9500
Zn2—N6^iv^	2.210 (3)	C30—H30	0.9500
Zn2—N5	2.269 (3)	C31—C32	1.528 (5)
O1—C31	1.253 (5)	C32—C33	1.526 (5)
O2—C31	1.268 (4)	C32—H32	1.0000
O2—Zn2^v^	2.052 (2)	C33—C34	1.500 (6)
O3—C37	1.254 (4)	C33—H33A	0.9900
O4—C37	1.254 (4)	C33—H33B	0.9900
O4—Zn1^vi^	2.080 (2)	C34—C35	1.547 (7)
N1—C1	1.332 (5)	C34—H34A	0.9900
N1—C5	1.344 (5)	C34—H34B	0.9900
N2—C6	1.338 (5)	C35—C36	1.462 (8)
N2—C10	1.340 (5)	C35—H35A	0.9900
N3—C15	1.337 (5)	C35—H35B	0.9900
N3—C11	1.338 (5)	C36—H36A	0.9900
N4—C20	1.325 (5)	C36—H36B	0.9900
N4—C16	1.348 (5)	C37—C38	1.541 (5)
N4—Zn1^iv^	2.242 (3)	C38—C39	1.522 (5)
N5—C21	1.335 (5)	C38—H38	1.0000
N5—C25	1.346 (5)	C39—C40	1.511 (6)
N6—C26	1.334 (5)	C39—H39A	0.9900
N6—C30	1.342 (5)	C39—H39B	0.9900
N6—Zn2^ii^	2.210 (3)	C40—C41	1.538 (7)
N7—C32	1.487 (5)	C40—H40A	0.9900
N7—H7A	0.9200	C40—H40B	0.9900
N7—H7B	0.9200	C41—C42	1.457 (7)
N8—C36	1.513 (7)	C41—H41A	0.9900
N8—H8A	0.9100	C41—H41B	0.9900
N8—H8B	0.9100	C42—H42A	0.9900
N8—H8C	0.9100	C42—H42B	0.9900
N9—C38	1.486 (4)	O1W—H1A	0.9547
N9—H9A	0.9200	O1W—H1B	0.8984
N9—H9B	0.9200	N11—O6	1.234 (6)
N10—C42	1.486 (6)	N11—O7	1.236 (6)
N10—H10A	0.9100	N11—O5	1.251 (6)
N10—H10B	0.9100	N12—O10	1.236 (5)
N10—H10C	0.9100	N12—O8	1.250 (5)
C1—C2	1.398 (6)	N12—O9	1.262 (5)
C1—H1	0.9500	N13—O12	1.230 (4)
C2—C3	1.379 (5)	N13—O13	1.239 (5)
C2—H2	0.9500	N13—O11	1.263 (5)
C3—C4	1.395 (5)	N14—O15	1.212 (6)
C3—C8	1.501 (4)	N14—O16	1.231 (6)
C4—C5	1.385 (5)	N14—O14	1.292 (6)
C4—H4	0.9500	O17—C43	1.233 (6)
C5—H5	0.9500	N15—C43	1.319 (6)
C6—C7	1.361 (5)	N15—C45	1.441 (6)
C6—H6	0.9500	N15—C44	1.444 (6)
C7—C8	1.389 (5)	C43—H43	0.9500
C7—H7	0.9500	C44—H44A	0.9800
C8—C9	1.385 (5)	C44—H44B	0.9800
C9—C10	1.387 (6)	C44—H44C	0.9800
C9—H9	0.9500	C45—H45A	0.9800
C10—H10	0.9500	C45—H45B	0.9800
C11—C12	1.382 (5)	C45—H45C	0.9800
C11—H11	0.9500	O18—C46	1.229 (9)
C12—C13	1.389 (5)	N16—C46	1.314 (7)
C12—H12	0.9500	N16—C47	1.444 (8)
C13—C14	1.387 (5)	N16—C48	1.463 (8)
C13—C18	1.488 (5)	C46—H46	0.9500
C14—C15	1.372 (5)	C47—H47A	0.9800
C14—H14	0.9500	C47—H47B	0.9800
C15—H15	0.9500	C47—H47C	0.9800
C16—C17	1.393 (5)	C48—H48A	0.9800
C16—H16	0.9500	C48—H48B	0.9800
C17—C18	1.401 (5)	C48—H48C	0.9800
C17—H17	0.9500	O18'—C46'	1.230 (9)
C18—C19	1.367 (5)	N16'—C46'	1.310 (8)
C19—C20	1.386 (5)	N16'—C47'	1.454 (9)
C19—H19	0.9500	N16'—C48'	1.461 (8)
C20—H20	0.9500	C46'—H46'	0.9500
C21—C22	1.378 (5)	C47'—H47D	0.9800
C21—H21	0.9500	C47'—H47E	0.9800
C22—C23	1.390 (5)	C47'—H47F	0.9800
C22—H22	0.9500	C48'—H48D	0.9800
C23—C24	1.383 (5)	C48'—H48E	0.9800
C23—C28	1.476 (5)	C48'—H48F	0.9800
			
O4^i^—Zn1—N7	101.75 (11)	C22—C23—C28	121.2 (3)
O4^i^—Zn1—O1	176.88 (10)	C25—C24—C23	120.3 (4)
N7—Zn1—O1	79.54 (11)	C25—C24—H24	119.8
O4^i^—Zn1—N1	87.76 (11)	C23—C24—H24	119.8
N7—Zn1—N1	170.06 (13)	N5—C25—C24	124.2 (4)
O1—Zn1—N1	91.12 (11)	N5—C25—H25	117.9
O4^i^—Zn1—N4^ii^	92.15 (10)	C24—C25—H25	117.9
N7—Zn1—N4^ii^	92.09 (13)	N6—C26—C27	124.2 (4)
O1—Zn1—N4^ii^	90.64 (10)	N6—C26—H26	117.9
N1—Zn1—N4^ii^	84.57 (12)	C27—C26—H26	117.9
O4^i^—Zn1—N3	87.85 (11)	C26—C27—C28	119.3 (4)
N7—Zn1—N3	93.47 (13)	C26—C27—H27	120.4
O1—Zn1—N3	89.23 (11)	C28—C27—H27	120.4
N1—Zn1—N3	89.76 (12)	C29—C28—C27	116.4 (3)
N4^ii^—Zn1—N3	174.33 (15)	C29—C28—C23	123.0 (3)
O2^iii^—Zn2—N9	102.08 (11)	C27—C28—C23	120.6 (4)
O2^iii^—Zn2—O3	179.04 (10)	C30—C29—C28	120.0 (4)
N9—Zn2—O3	77.21 (11)	C30—C29—H29	120.0
O2^iii^—Zn2—N2	91.85 (11)	C28—C29—H29	120.0
N9—Zn2—N2	166.03 (12)	N6—C30—C29	123.9 (4)
O3—Zn2—N2	88.87 (11)	N6—C30—H30	118.0
O2^iii^—Zn2—N6^iv^	88.14 (10)	C29—C30—H30	118.0
N9—Zn2—N6^iv^	94.08 (13)	O1—C31—O2	125.6 (3)
O3—Zn2—N6^iv^	91.26 (10)	O1—C31—C32	119.8 (3)
N2—Zn2—N6^iv^	87.23 (12)	O2—C31—C32	114.5 (4)
O2^iii^—Zn2—N5	87.49 (10)	N7—C32—C33	113.9 (3)
N9—Zn2—N5	91.50 (12)	N7—C32—C31	110.9 (3)
O3—Zn2—N5	93.16 (10)	C33—C32—C31	111.3 (3)
N2—Zn2—N5	88.14 (12)	N7—C32—H32	106.8
N6^iv^—Zn2—N5	173.53 (15)	C33—C32—H32	106.8
C31—O1—Zn1	116.2 (2)	C31—C32—H32	106.8
C31—O2—Zn2^v^	129.8 (3)	C34—C33—C32	116.0 (4)
C37—O3—Zn2	115.8 (2)	C34—C33—H33A	108.3
C37—O4—Zn1^vi^	129.5 (2)	C32—C33—H33A	108.3
C1—N1—C5	117.8 (3)	C34—C33—H33B	108.3
C1—N1—Zn1	122.4 (3)	C32—C33—H33B	108.3
C5—N1—Zn1	119.5 (3)	H33A—C33—H33B	107.4
C6—N2—C10	116.7 (3)	C33—C34—C35	110.8 (4)
C6—N2—Zn2	119.9 (2)	C33—C34—H34A	109.5
C10—N2—Zn2	123.2 (3)	C35—C34—H34A	109.5
C15—N3—C11	115.7 (3)	C33—C34—H34B	109.5
C15—N3—Zn1	122.1 (3)	C35—C34—H34B	109.5
C11—N3—Zn1	121.9 (2)	H34A—C34—H34B	108.1
C20—N4—C16	116.2 (3)	C36—C35—C34	113.2 (5)
C20—N4—Zn1^iv^	124.8 (2)	C36—C35—H35A	108.9
C16—N4—Zn1^iv^	118.7 (2)	C34—C35—H35A	108.9
C21—N5—C25	115.2 (3)	C36—C35—H35B	108.9
C21—N5—Zn2	120.0 (2)	C34—C35—H35B	108.9
C25—N5—Zn2	124.8 (2)	H35A—C35—H35B	107.7
C26—N6—C30	116.1 (3)	C35—C36—N8	111.5 (5)
C26—N6—Zn2^ii^	122.1 (3)	C35—C36—H36A	109.3
C30—N6—Zn2^ii^	121.8 (3)	N8—C36—H36A	109.3
C32—N7—Zn1	111.6 (2)	C35—C36—H36B	109.3
C32—N7—H7A	109.3	N8—C36—H36B	109.3
Zn1—N7—H7A	109.3	H36A—C36—H36B	108.0
C32—N7—H7B	109.3	O3—C37—O4	124.9 (3)
Zn1—N7—H7B	109.3	O3—C37—C38	118.9 (3)
H7A—N7—H7B	108.0	O4—C37—C38	116.1 (3)
C36—N8—H8A	109.5	N9—C38—C39	115.5 (3)
C36—N8—H8B	109.5	N9—C38—C37	108.1 (3)
H8A—N8—H8B	109.5	C39—C38—C37	113.9 (3)
C36—N8—H8C	109.5	N9—C38—H38	106.2
H8A—N8—H8C	109.5	C39—C38—H38	106.2
H8B—N8—H8C	109.5	C37—C38—H38	106.2
C38—N9—Zn2	110.6 (2)	C40—C39—C38	112.9 (4)
C38—N9—H9A	109.5	C40—C39—H39A	109.0
Zn2—N9—H9A	109.5	C38—C39—H39A	109.0
C38—N9—H9B	109.5	C40—C39—H39B	109.0
Zn2—N9—H9B	109.5	C38—C39—H39B	109.0
H9A—N9—H9B	108.1	H39A—C39—H39B	107.8
C42—N10—H10A	109.5	C39—C40—C41	113.7 (5)
C42—N10—H10B	109.5	C39—C40—H40A	108.8
H10A—N10—H10B	109.5	C41—C40—H40A	108.8
C42—N10—H10C	109.5	C39—C40—H40B	108.8
H10A—N10—H10C	109.5	C41—C40—H40B	108.8
H10B—N10—H10C	109.5	H40A—C40—H40B	107.7
N1—C1—C2	122.3 (4)	C42—C41—C40	115.8 (4)
N1—C1—H1	118.8	C42—C41—H41A	108.3
C2—C1—H1	118.8	C40—C41—H41A	108.3
C3—C2—C1	119.8 (4)	C42—C41—H41B	108.3
C3—C2—H2	120.1	C40—C41—H41B	108.3
C1—C2—H2	120.1	H41A—C41—H41B	107.4
C2—C3—C4	117.8 (4)	C41—C42—N10	116.0 (5)
C2—C3—C8	121.7 (3)	C41—C42—H42A	108.3
C4—C3—C8	120.5 (3)	N10—C42—H42A	108.3
C5—C4—C3	118.9 (4)	C41—C42—H42B	108.3
C5—C4—H4	120.5	N10—C42—H42B	108.3
C3—C4—H4	120.5	H42A—C42—H42B	107.4
N1—C5—C4	123.2 (4)	H1A—O1W—H1B	106.0
N1—C5—H5	118.4	O6—N11—O7	122.3 (7)
C4—C5—H5	118.4	O6—N11—O5	119.3 (7)
N2—C6—C7	123.9 (4)	O7—N11—O5	118.2 (5)
N2—C6—H6	118.0	O10—N12—O8	121.6 (4)
C7—C6—H6	118.0	O10—N12—O9	120.6 (4)
C6—C7—C8	120.0 (4)	O8—N12—O9	117.7 (4)
C6—C7—H7	120.0	O12—N13—O13	120.6 (4)
C8—C7—H7	120.0	O12—N13—O11	119.7 (4)
C9—C8—C7	116.8 (3)	O13—N13—O11	119.8 (4)
C9—C8—C3	122.2 (3)	O15—N14—O16	123.0 (7)
C7—C8—C3	121.0 (3)	O15—N14—O14	120.5 (6)
C10—C9—C8	119.8 (4)	O16—N14—O14	115.8 (6)
C10—C9—H9	120.1	C43—N15—C45	120.1 (5)
C8—C9—H9	120.1	C43—N15—C44	120.4 (5)
N2—C10—C9	122.8 (4)	C45—N15—C44	119.5 (5)
N2—C10—H10	118.6	O17—C43—N15	123.5 (6)
C9—C10—H10	118.6	O17—C43—H43	118.3
N3—C11—C12	124.1 (4)	N15—C43—H43	118.3
N3—C11—H11	118.0	N15—C44—H44A	109.5
C12—C11—H11	118.0	N15—C44—H44B	109.5
C11—C12—C13	119.3 (4)	H44A—C44—H44B	109.5
C11—C12—H12	120.3	N15—C44—H44C	109.5
C13—C12—H12	120.3	H44A—C44—H44C	109.5
C14—C13—C12	116.8 (3)	H44B—C44—H44C	109.5
C14—C13—C18	122.2 (3)	N15—C45—H45A	109.5
C12—C13—C18	121.0 (4)	N15—C45—H45B	109.5
C15—C14—C13	119.7 (3)	H45A—C45—H45B	109.5
C15—C14—H14	120.2	N15—C45—H45C	109.5
C13—C14—H14	120.2	H45A—C45—H45C	109.5
N3—C15—C14	124.2 (4)	H45B—C45—H45C	109.5
N3—C15—H15	117.9	C46—N16—C47	119.0 (7)
C14—C15—H15	117.9	C46—N16—C48	121.0 (8)
N4—C16—C17	123.5 (4)	C47—N16—C48	119.7 (7)
N4—C16—H16	118.2	O18—C46—N16	123.2 (10)
C17—C16—H16	118.2	O18—C46—H46	118.4
C16—C17—C18	118.6 (4)	N16—C46—H46	118.4
C16—C17—H17	120.7	C46'—N16'—C47'	120.5 (8)
C18—C17—H17	120.7	C46'—N16'—C48'	121.3 (9)
C19—C18—C17	117.7 (3)	C47'—N16'—C48'	118.2 (8)
C19—C18—C13	121.5 (4)	O18'—C46'—N16'	123.6 (11)
C17—C18—C13	120.8 (3)	O18'—C46'—H46'	118.2
C18—C19—C20	119.6 (4)	N16'—C46'—H46'	118.2
C18—C19—H19	120.2	N16'—C47'—H47D	109.5
C20—C19—H19	120.2	N16'—C47'—H47E	109.5
N4—C20—C19	124.3 (4)	H47D—C47'—H47E	109.5
N4—C20—H20	117.8	N16'—C47'—H47F	109.5
C19—C20—H20	117.8	H47D—C47'—H47F	109.5
N5—C21—C22	124.2 (4)	H47E—C47'—H47F	109.5
N5—C21—H21	117.9	N16'—C48'—H48D	109.5
C22—C21—H21	117.9	N16'—C48'—H48E	109.5
C21—C22—C23	120.0 (4)	H48D—C48'—H48E	109.5
C21—C22—H22	120.0	N16'—C48'—H48F	109.5
C23—C22—H22	120.0	H48D—C48'—H48F	109.5
C24—C23—C22	116.0 (3)	H48E—C48'—H48F	109.5
C24—C23—C28	122.9 (4)		

Symmetry codes: (i) −*x*+1, *y*−1/2, −*z*+1; (ii) *x*, *y*, *z*+1; (iii) −*x*+2, *y*+1/2, −*z*+1; (iv) *x*, *y*, *z*−1; (v) −*x*+2, *y*−1/2, −*z*+1; (vi) −*x*+1, *y*+1/2, −*z*+1.

### Hydrogen-bond geometry (Å, º)

**Table:**

*D*—H···*A*	*D*—H	H···*A*	*D*···*A*	*D*—H···*A*
O1*W*—H1*A*···O15	0.95	1.91	2.823 (11)	159
O1*W*—H1*B*···O10^ii^	0.90	2.48	3.182 (8)	136
N7—H7*A*···O3^i^	0.92	2.54	3.066 (4)	117
N7—H7*B*···O18	0.92	2.03	2.940 (9)	167
N7—H7*B*···O18′	0.92	2.32	3.215 (17)	163
N8—H8*A*···O17^vii^	0.91	1.86	2.755 (7)	166
N8—H8*B*···O8^vii^	0.91	2.04	2.842 (6)	147
N8—H8*B*···O9^vii^	0.91	2.39	3.057 (6)	130
N8—H8*C*···O5^vii^	0.91	2.00	2.907 (7)	172
N9—H9*A*···O1^iii^	0.92	2.44	2.999 (4)	119
N9—H9*B*···O10	0.92	2.16	3.075 (4)	174
N10—H10*A*···O14	0.91	2.00	2.869 (6)	160
N10—H10*A*···O16	0.91	2.44	3.183 (8)	139
N10—H10*B*···O11^viii^	0.91	2.00	2.844 (5)	154
N10—H10*B*···O12^viii^	0.91	2.48	3.066 (6)	123
N10—H10*C*···O7^ii^	0.91	2.04	2.914 (7)	161

Symmetry codes: (i) −*x*+1, *y*−1/2, −*z*+1; (ii) *x*, *y*, *z*+1; (iii) −*x*+2, *y*+1/2, −*z*+1; (vii) *x*, *y*−1, *z*; (viii) *x*, *y*+1, *z*.
